# Metastatic pituitary carcinoma in a patient with acromegaly: a case report

**DOI:** 10.1186/1752-1947-6-322

**Published:** 2012-09-25

**Authors:** Seamus Sreenan, Elizabeth Sengupta, William Tormey, Richard Landau

**Affiliations:** 1 Department of Endocrinology, Connolly Hospital, Dublin 15, Ireland; 2 The Royal College of Surgeons in Ireland, Dublin 2, Ireland; 3 Department of Pathology, University of Chicago, Medical Center, Chicago, IL, USA; 4 Department of Chemical Pathology, Connolly Hospital, Dublin 15, Ireland; 5 University of Ulster at Coleraine, Coleraine, Northern Ireland; 6 Department of Endocrinology, University of Chicago Medical Center, Chicago, IL, USA

**Keywords:** Metastatic pituitary carcinoma, Acromegaly

## Abstract

**Introduction:**

Asymptomatic pituitary abnormalities occur in about 10% of cranial magnetic resonance imaging scans, but metastatic carcinoma of the pituitary gland is rare: 133 cases have been reported. Two thirds secreted either prolactin or adrenocorticotropic hormone, and another 24% were non-secreting.

**Case presentation:**

A 42-year-old Caucasian man lived for 30 years after the diagnosis of a pituitary tumor whose clinical and biochemical features were those of acromegaly and hypogonadism. Radiotherapy, totaling 7300 rad, was administered to the sella over two courses. Growth hormone levels normalized, but he developed both thyroid and adrenal insufficiency, and replacement therapy was commenced. Fourteen years later, growth hormone levels again became elevated, and bromocriptine was commenced but led to side effects that could not be tolerated. An attempted surgical intervention failed, and octreotide and pergolide were used in succession. Twenty-seven years after the diagnosis, a mass from an excisional biopsy of below the angle of the mandible proved to be metastatic pituitary carcinoma. Immunohistochemical staining was positive for synaptophysin, growth hormone, and prolactin. One year later, an octreotide scan showed uptake at the sella, neck, and spleen. Our patient declined further active oncology treatment.

**Conclusions:**

Metastatic pituitary carcinoma associated with acromegaly is particularly rare. To the best of our knowledge, this is the eighth such case and is the first report of growth hormone and prolactin present in the metastatic mass.

## Introduction

Magnetic resonance imaging scans show that about 10% of the healthy adult population have pituitary abnormalities, which are likely asymptomatic pituitary adenomas [1]. Most pituitary tumors are benign adenomas, and adenocarcinomas are rare. A case report and review in 2011 [2] showed that 132 cases of pituitary carcinoma had been recorded in 50 years. Growth hormone (GH) was elevated in only seven cases, and prolactin was also raised in one of these [3-9]. Acromegaly was the clinical picture in three [3,8,9]. Visual impairment [4], headache [5], and amenorrhea [7] were the chief symptoms in another three, and no symptom details are available in one case [6]. We report a further case of acromegaly with metastatic pituitary carcinoma.

## Case presentation

A 42-year-old Caucasian man complained of headache, vomiting, and blurred vision. A pituitary tumor was diagnosed after a skull X-ray, and he received 3300 rad to his sella. Two years later, he presented with impotence, joint pains, and perspiration. His serum GH level was 16.2μg/L (reference range is 0.45 to 2.2) and was not suppressed by the administration of oral glucose. His serum testosterone level was subnormal at 423nmol/L (reference range is 797 to 3470). His visual fields were normal, but a skull X-ray demonstrated an enlarged sella. Acromegaly with partial hypopituitarism was diagnosed. Further radiation therapy (4000 rad) was administered, and he received testosterone replacement. His GH levels were normalized, but 3 years later hypothyroidism and hypoadrenalism were diagnosed, and appropriate replacement therapies were begun.

Fourteen years after the initial diagnosis, his GH levels again became elevated (25μg/L). A computed tomography scan revealed a partially empty sella but no tumor. He received therapy with up to 5mg of bromocriptine twice daily for 1 year and then the drug was discontinued because of intolerable side effects (nausea, headache, and diarrhea). Surgical exploration was deemed inappropriate because of previous irradiation, especially in the absence of radiologic evidence of a pituitary mass. After 18 years, a computed tomography scan, which was performed because of progressive enlargement of the hands, feet, and nose and an elevation of the GH level (104.5μg/L), revealed a sellar mass. Attempted transsphenoidal exploration was abandoned as a result of failed intubation.

Therapy with octreotide by subcutaneous injection was initiated. The dose was gradually increased to the maximum approved level of 500mg three times daily and was administered by continuous subcutaneous infusion. GH levels were reduced to 14.5μg/L but not normalized. After four more years, octreotide was discontinued and therapy with pergolide commenced. However, GH levels continued to increase.

Twenty-seven years into the disease course, the patient developed a soft, painless, 4cm mass below the angle of his right mandible. Fine-needle aspiration was suspicious for lymphoma, and an excisional biopsy was performed.

**Figure 1:**
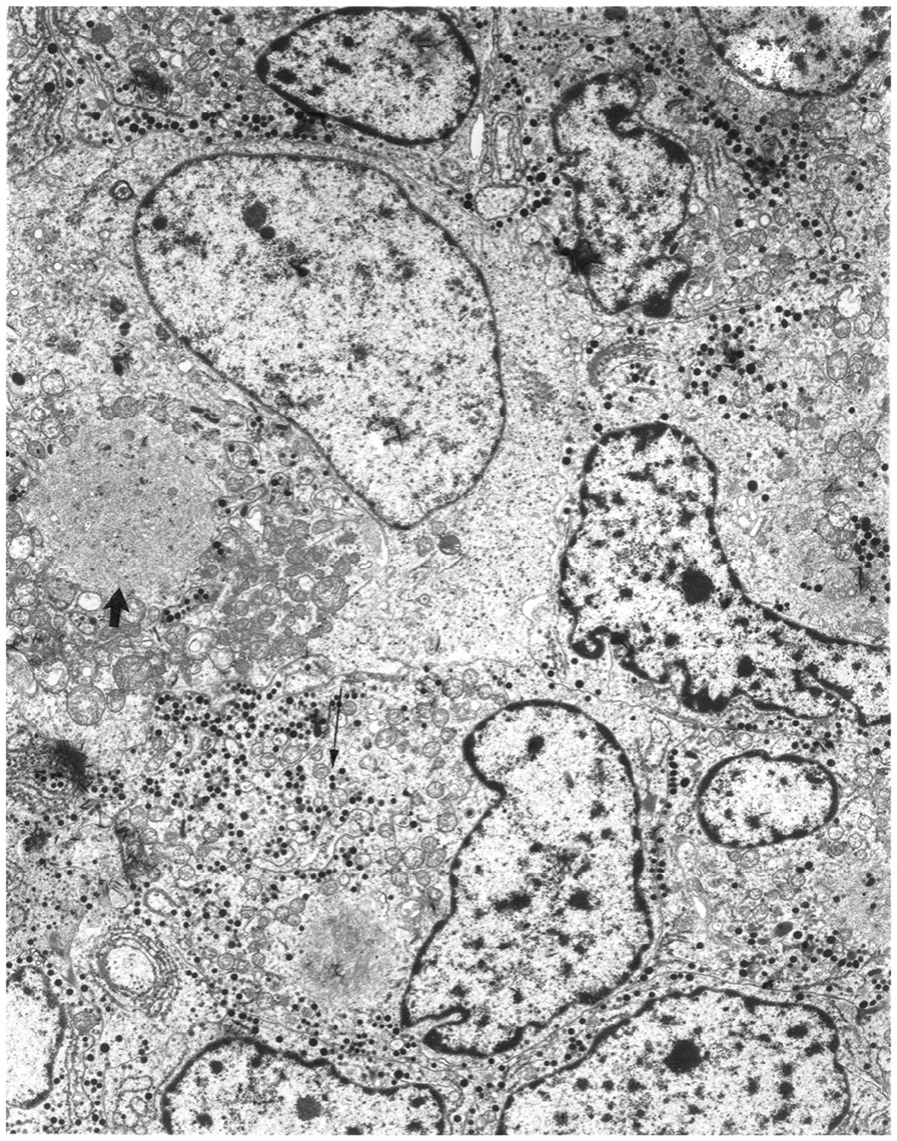
** Photomicrograph of a section of the mass removed from the patient’s neck.** The numerous dense core granules (long arrow) indicate that the mass originated from neuroendocrine tissue. Two globular fibrous bodies are also present in the section (one marked with the short arrow). The bodies are suggestive of a growth hormone-producing tumor.

Histopathology revealed tumor cells with abundant pink cytoplasm. Electron microscopy demonstrated cells containing membrane-bound dense core granules indicative of a neuroendocrine tumor. Some cells contained globular fibrous bodies of intermediate filaments suggestive of a GH-secreting tumor (Figure 1). Immunohistochemical staining was positive for synaptophysin, GH, and prolactin but negative for other anterior pituitary hormones. A diagnosis of metastatic pituitary carcinoma was made.

The GH level decreased from 363μg/L before surgery to 101μg/L but on subsequent follow-up began to rise again. One year later, a radionuclide scan with indium-labeled octreotide showed continued uptake in the region of the sella, neck, and spleen. The patient declined further investigation or active treatment for acromegaly and was treated symptomatically before he died, at the age of 72, 30 years after initial presentation.

## Discussion

Pituitary carcinomas may be non-functioning or hormone-secreting. In the review series on 132 pituitary carcinomas [2], 23.5% were non-functioning, 36.4% secreted prolactin only, 29.5% secreted adrenocorticotropic hormone only, and 0.2% secreted gonadotropin-releasing hormone; there were individual cases of thyroid-stimulating hormone (TSH) and luteinizing hormone (LH) secretion and single cases of prolactin and LH secretion and prolactin and TSH secretion. In the GH cases, there were six women and this report is the second man. The age at presentation in the GH cases ranged from 19 to 48 years, and the median age was 42 years. The commonest metastatic site for these cases was the lymph nodes (in 50% of cases). Spinal and intracranial sites were also reported as sites of metastases. The present case is unique in the extent of proven and potential metastases. Treatment modalities include surgery, radiotherapy, bromocriptine, somatostatin analogs, and chemotherapy. The duration of follow-up in these cases ranged from 48 to 192 months and in this case was 27 years. The clinical picture at the time of reporting was that three were stable and three had a partial response to treatment; no information is available in the remaining case. No postmortem information is available in any of these cases.

Treatment of malignant pituitary tumors includes surgery to remove the primary and metastatic tumor augmented by conventional and stereotactic radiotherapy. The mode of radiation depends on the availability of technology with Gamma Knife^®^, linear accelerator, and proton beam used. The relative value of stereotactic radiosurgery in malignant cases awaits investigation. The role of treatments with dopamine agonists cabergoline or bromocriptine and somatostatin analogs such as lanreotide and octreotide is case-dependent, but follow-up should continue for life. Drugs that block GH receptors, such as pegvisomant, may relieve symptoms but do not reduce the tumor size and should not be used in malignant cases.

GH production by the cervical metastasis was not unequivocally demonstrated, but reduction in GH following surgical removal of the neck mass suggests that the continued GH production was originating, at least in part, from the extracranial metastasis. Prolactin levels were not measured for many years prior to the removal of the metastasis and were subsequently undetectable. However, the positive immunostaining for prolactin in addition to GH in an extracranial metastasis from a GH-producing tumor is a unique feature of the case [7].

The etiology of pituitary carcinoma is poorly understood. It has been suggested that radiation may induce mutations in tumor cells, making them more likely to metastasize. A number of cases of sarcomas arising many years after cranial irradiation in tissues previously exposed to radiation have been reported [10,11]. High-dose radiation therapy, particularly if given (as in this case) in divided doses, has been implicated in their pathogenesis. Our patient’s total radiation dose over the course of 3 years was 7300 rad. Similarly, all but one [9] of the other patients with GH-secreting pituitary carcinoma had received cranial irradiation for their primary tumors. Radiotherapy is standard therapy in acromegaly and is regarded as relatively safe in current modes. Stereotactic radiotherapy, when available, is standard [12]. An overview of pituitary carcinoma found that a Ki-67 level of above 10% suggests increased metastatic potential in pituitary tumors. Ki-67 is found in all active phases of the cell cycle but not in resting cells. The roles of p53, retinoblastoma gene, nm23 protein, H-ras oncogene, galectin-3, Her-2/neu protooncogene, cyclooxygenase-2, and activated epidermal growth factor receptor in pituitary tumors are also summarized [13].

## Conclusions

Metastatic pituitary carcinoma associated with acromegaly is rare. To the best of our knowledge, this is the eighth case and the first report of GH and prolactin present in the metastatic mass.

## Consent

Despite all reasonable attempts, written informed consent for publication from the patient’s next of kin could not be obtained. The case is important to public health, and every effort has been made to protect the identity of our patient. There is no reason to believe that our patient’s next of kin would object to publication.

## Abbreviations

GH: Growth Hormone; LH: Luteinizing Hormone; TSH: Thyroid-Stimulating Hormone.

## Competing interests

The authors declare that they have no competing interests.

## Authors’ contributions

SS and RL managed the patient clinically, ES reported the histology and WT wrote the text. All authors read and approved the final manuscript.
